# L- shape symphyseal autogenous bone block for alveolar ridge augmentation in anterior maxilla and mandible, a prospective single arm clinical study

**DOI:** 10.1186/s12903-025-05601-6

**Published:** 2025-03-01

**Authors:** Wael Mohamed Said Ahmed, Ahmed F. Arafa

**Affiliations:** 1 Department of Oral and Maxillofacial Surgery, Faculty of Dentistry, Mansoura University, Mansoura, Egypt; 2 Oral and Maxillofacial Resident, Oral and Maxillofacial Department, Zagazig University, Zagazig, 44519 Egypt

**Keywords:** Bone grafting, Ridge augmentation, Maxillary, Mandibular

## Abstract

**Background:**

Alveolar ridge augmentations are challenging procedures in dental implantology, especially in esthetic zone. 3D alveolar defects can be treated by guided bone regeneration (GBR), distraction osteogenesis, or bone blocks. This study introduces a new technique for 3D-alveolar ridge augmentation by using L-shape autogenous symphyseal bone block.

**Purpose:**

This study aimed to assess both horizontal and vertical alveolar bone augmentation for severe atrophied anterior maxilla and mandible, using an L- shape autogenous bone block harvested from the symphysis.

**Patients and method:**

elven partially edentulous patients who needed horizontal and vertical bone augmentation in the anterior maxilla or mandible before implant placement were selected for this study. For each patient, an autogenous bone block was harvested from the symphysis, trimmed to L-shape, and used to augment the anterior maxilla or mandible horizontally and vertically. Horizontal and vertical bone gain was measured by CBCT immediate postoperative and at 6months postoperatively.

**Results:**

In this study, 14 L-shape bone blocks were grafted in 11 patients. The patients were 4males and 7females, with a mean age of 24.63years. Healing was uneventful for all patients with no sensory disturbance. The Mean of horizontal bone gain was 4.17 ± 0.77 mm immediate postoperative, and was 3.52 ± 0.75 after 6months. While, the mean of vertical bone gain was 6.51 ± 1.01 mm immediate postoperative, and was 4.74 ± 1.03after 6months. The mean of horizontal and vertical bone loss was 0.74 ± 0.24 mm and 1.62 ± 0.19 mm after 6 months, respectively.

**Conclusion:**

Using L- shape autogenous bone block harvested from the symphysis for alveolar ridge augmentation is a safe, predictable and effective method for 3D ridge augmentation.

## Introduction

Ideal implant installation is frequently complicated with atrophic alveolar ridge, particularly in the aesthetic zone. Horizontal and/or vertical bone loss occurs mostly after tooth extraction. Tan et al. [1] concluded in their systematic review, after tooth extraction there was a rapid resorption rate of the alveolar bone in the first 3–6 months followed by gradual resorption, with 29–63% horizontal bone loss and 11–22% vertical bone loss after 6months.

Both horizontal and vertical(3D) alveolar ridge augmentations are challenging procedures in dental implantology [2]. If an optimal 3D placement of implants cannot be achieved in the residual alveolar bone, ridge augmentation should be performed. Ridge augmentation reestablishes sufficient alveolar bone volume for proper implant insertion, consequently, this enhances aesthetic outcomes, restores inter-arch relation, improves osteointegration and increases implant survival [3]. 3D defects can be treated by guided bone regeneration (GBR), distraction osteogenesis, or bone blocks [4].

Although GBR is a widely used augmentation method, it cannot attain significant bone height, therefore it is commonly used for minor deficiencies [5, 6]. Distraction osteogenesis is the most commonly used modality capable of bone gain that may exceed 10 mm in height and 6 mm in width. Unfortunately, it has some drawbacks including device exposure, infection, high cost, and being a difficult technique [6–8].

Autogenous bone graft is considered the gold standard in ridge augmentation, and it remains one of the main methods for alveolar ridge reconstruction [3, 9]. These block grafts can be harvested either from intraoral or extraoral sites. Intraoral bone grafts (membranous bone grafts, such as symphysis and mandibular ramus) show less resorption rate and faster revascularization compared to extraoral ones (endochondral bone grafts, such as iliac crest and ribs and membranous bone grafts as calvarial bone graft, which is less resorbable than other extraoral bone grafts) [9]. Furthermore, intraoral bone blocks can be harvested under local anesthesia with less complications [10].

Khoury et al. [11] in their popular bone shell technique for 3D ridge augmentation, split the mandibular bone block into two thin plates, which is a difficult manoeuvre. This technique involves the reflection of both labial and palatal flaps to fix the two bone shells, which may result in encroaching the nasopalatine nerve in anterior maxilla. Furthermore, a bone scraper was used to harvest autogenous bone chips from the donor site to fill the gap between the buccal and palatal bone shells. In a different technique, two separate bone blocks have been used to augment deficient alveolar ridges; one block is fixed buccally with at least two screws and the other one is fixed on the alveolar crest by additional two screws [2]. However, when the crest of the alveolar ridge is thin, the stability of the crestal bone block may be questionable affecting the augmentation outcomes. To overcome these limitations, L- shape bone block was used in this study and was fixed with only two horizontal screws. So, this study aimed to assess 3D alveolar ridge augmentation for sever atrophied anterior maxilla and mandible using L- shape autogenous bone block grafts harvested from the symphysis.

## Patients and method

This prospective single arm clinical study was approved by the Ethical Committee of the Faculty of Dentistry, Mansoura University(A015010222), and the guidelines of Helsinki Declaration were followed. The study was conducted from February/2021 to August/2022, and according to STROBE guidelines for clinical trials. This study was registered in Clinicaltrials. gov with ID (NCT05844540) on 06/05/2023. Elven partially edentulous patients who needed horizontal and vertical bone augmentation in the anterior maxilla or mandible before implant placement were included in this study. The patients were recruited from the Outpatient Clinic of the Oral and Maxillofacial Department, Faculty of Dentistry, Mansoura University according to the following:

### Inclusion criteria

(1) Missed one or more upper or lower anterior teeth; (2) alveolar ridge with horizontal and vertical bone loss (class III according to Seibert et al. [12] classification); (3) age 18 to 45 years; and (4) healthy oral mucosa, at least 3 mm keratinized mucosa.

### Exclusion criteria

(1) Poor interest and non-cooperated patients; (2) patients with disturbed occlusion, and inadequate inter-arch space; (3) patients with systemic factors that affect bone healing as uncontrolled diabetes, osteoporosis, immune deficiency, chemotherapy and anti-bone resorptive medications (bisphosphonates); (4) patients with local factors that affect bone healing as a history of radiation therapy, and presence of local bone pathology(cyst or tumour); (5) patients with parafunction habits as bruxism or clenching; (6) pregnancy; (7) smokers; and (8) poor oral hygiene.

Before the surgical operation, all patients were informed about the treatment options, the nature of the operation, and the probable postoperative sequelae. An informed consent was signed from each patient.

### Preoperative assessment

- Patients’ medical status were carefully evaluated.
- A thorough intraoral examination was done to evaluate oral hygiene with mechanical plaque removal if needed, occlusion, and mucosa that covers the recipient and donor sites.
- Preoperative CBCT was taken for each patient to evaluate bone width and height in both the recipient and donor sites.

### Surgical procedures

Prophylactic antibiotic 600 mg Clindamycin (Clindam, Sigma, Egypt) was administered 1 h before surgery. A thorough mouth rinsing using Chlorhexidine mouth wash (Hexitol, Adco, Egypt) for about 1 min was done immediately before surgery.

### Harvesting procedure of the symphysis bone block

**Fig. 1:**
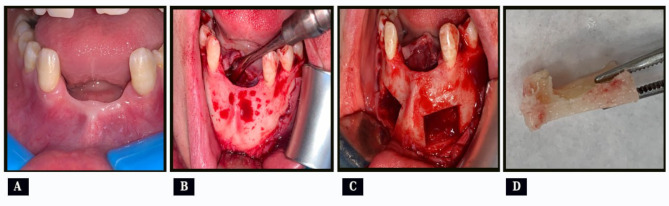
(case#3): **A**, Preoperative intraoral photograph; **B**, Exposure of the symphysis after elevation of full thickness mucoperiosteal flap; **C**, Symphysis after harvesting rectangular bone blocks; **D**, L-shape bone block

Under local anesthesia, a sulcular incision was performed from lower right to left second premolars with an oblique incision distal to each one. After that, a full thickness mucoperiosteal flap was elevated to expose the anterior mandible including the entire symphysis and both mental nerves (Fig. 1, A&B). By using piezo-surgery unit (Surgic Touch, Woodpecker, China), a rectangular bone block was harvested from the symphysis about 8 mm in depth and 2 mm wider than the recipient site to allow for contouring. The bone cuts were performed 5 mm far from the apices of the anterior teeth, mental foramina, and inferior border of the mandible (Fig. 1, C) [13]. Finally, thin curved osteotomes were used to detach the bone blocks. The harvested bone block was trimmed to form L-shape block and kept in normal saline (Fig. 1, D). After hemostasis, the flap was closed with 4/0 vicryl interrupted sutures.

### Recipient site preparation

**Fig. 2:**
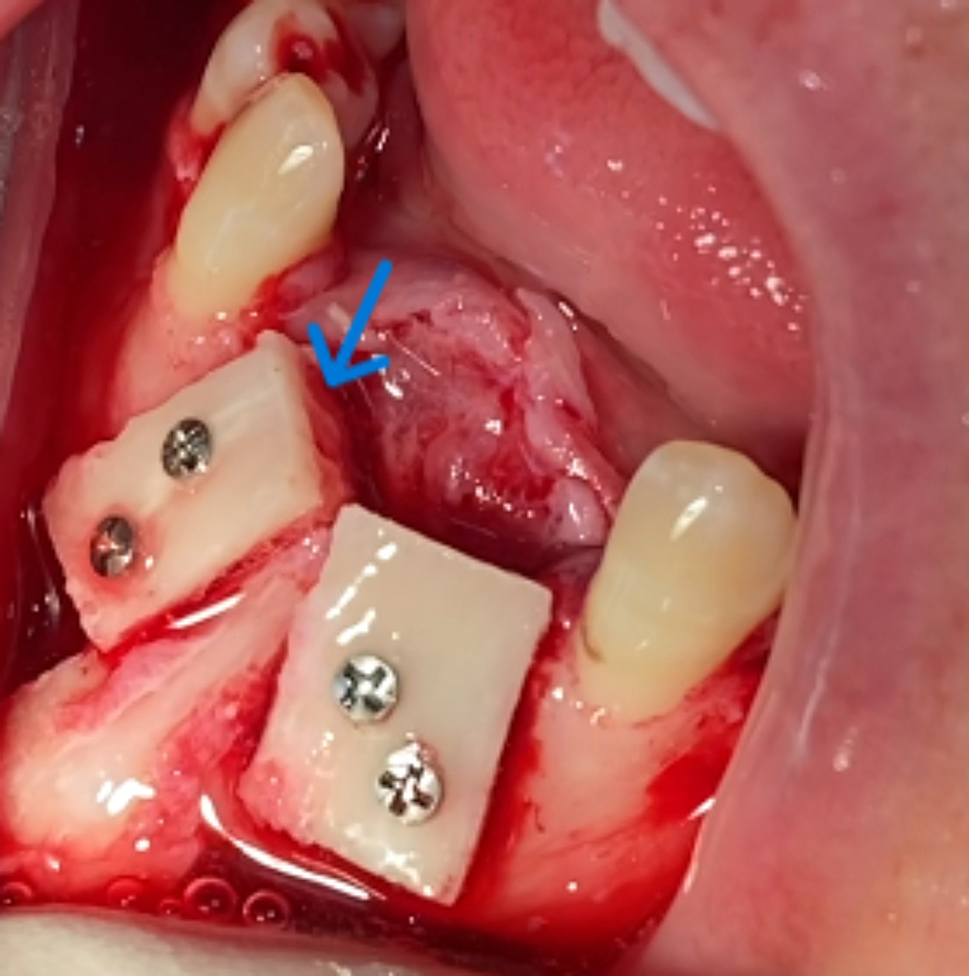
(case#3): Fixation of L-shape bone blocks (right and left) with two 1.5 mm screws for each. The arrow refers to the horizontal arm of the right L-shape bone block

At the maxillary recipient site, after induction of local anesthesia, a mid-crestal incision that continued in the sulcus for two teeth on either side of the defect with bilateral oblique incisions were performed. After that, the full thickness mucoperiosteal flap was elevated. While for mandibular recipient site, it was the same flap used for harvesting the symphyseal bone block. For both upper or lower recipient sites, perforations (bone marrow penetration) were done to the recipient site by using a fissure surgical bur. The L-shape block graft was trimmed to obtain optimal adaptation to the recipient site, then it was fixed to the residual ridge with two 1.5 mm and 9 mm long self-tapping titanium screws. (Fig. 2). After achieving graft stability, any sharp edges were smoothed and any gap between the graft and underlying alveolar bone was filled with particulate bone curetted from the donner site. Finally, the flap was relaxed by periosteal incisions and blunt muscle dissection from the anterior maxilla or mandible to obtain a primary tension free closure using 4/0 vicryl interrupted and horizontal mattress sutures.

### Postsurgical instructions

Patients were instructed to avoid any wound trauma, and apply ice packs on the host and recipient surgical sites 20 min/ hour for the first day after surgery. For maintenance of proper oral hygiene mouth rinsing with chlorhexidine mouth wash was prescribed 3times/ day for 1 week. Diclofenac potassium 50 mg (Cataflam, Novartis, Switzerland) anti-inflammatory analgesic was prescribed as needed. Clindamycin 300 mg twice daily was prescribed for 7days.

### Temporary prosthesis

**Fig. 3:**
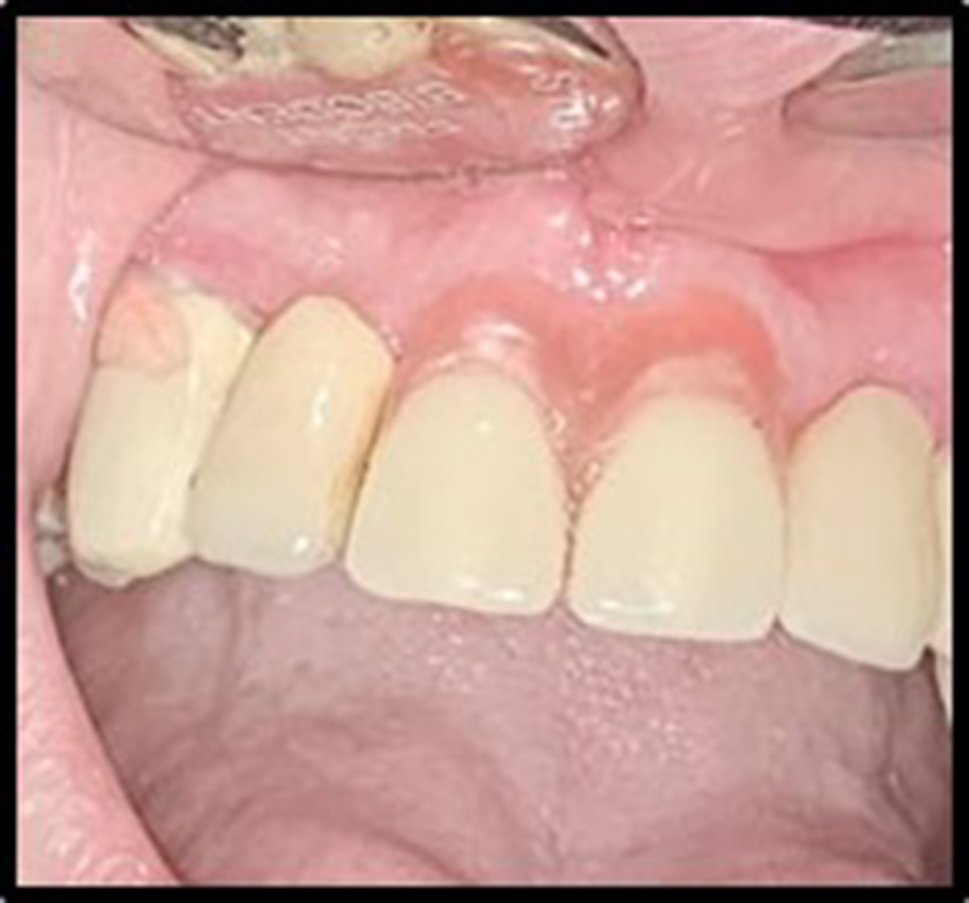
(case#10): A flexible partial denture as a temporary prosthesis

A flexible partial denture as a temporary prosthesis was made for each patient one month after the surgery. (Fig. 3)

### Implant insertion

**Fig. 4:**
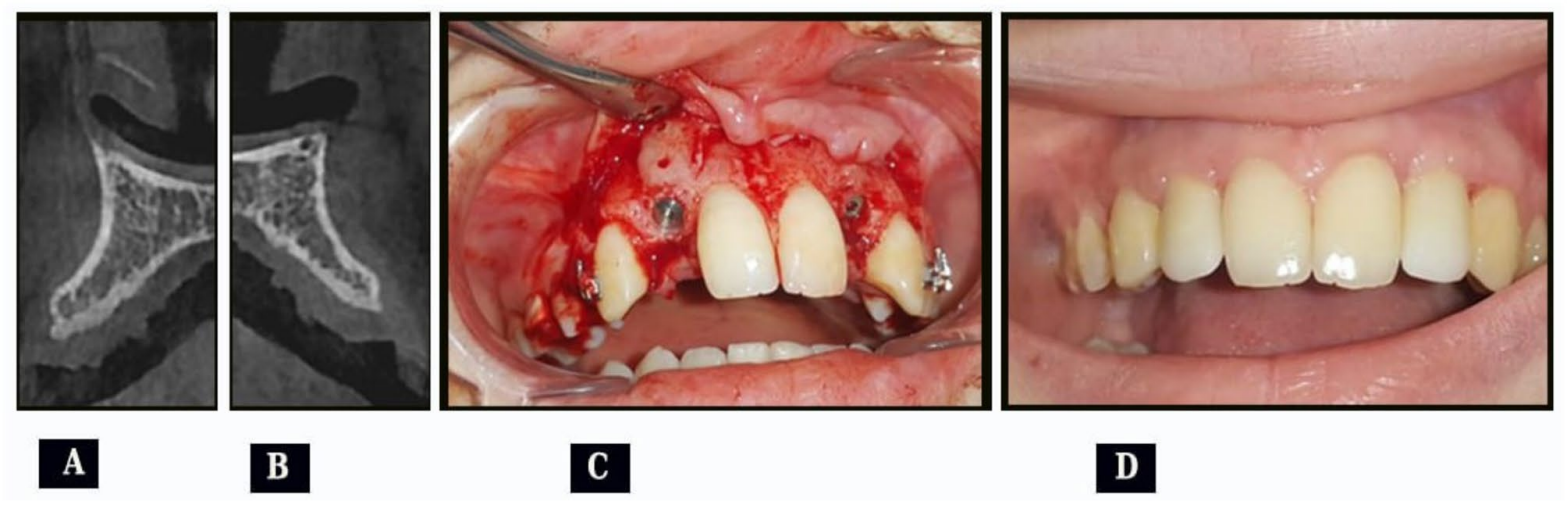
(case#6): **A&B**, Preoperative CBCT cross sectional view showing atrophied right (**A**) and left (**B**) alveolar ridges; **(C)**, Removal of fixation screws and implant insertion; (**D**), Final restoration

After 6months, the surgical site was opened to remove the fixation screws and to insert dental implants. The final restoration was done 4months after implant insertion. (Fig. 4)

#### Evaluation

the patients were scheduled at 3days, 14days, 3months, and 6months postoperatively to evaluate:

#### Postoperative pain

Pain intensity was measured at three days after surgery on a 0 to 10 numerical rating scale in which 0 = no pain, 1–3 = mild pain, 4–6 = moderate pain, 7–9 = severe pain, and 10 = the worst pain imaginable.

#### Soft tissue healing

The wound healing index of Landry et al. (14) was utilized to assess the soft tissue healing over the grafted area after 14 days from surgery. The index classifies the healing pattern into: very poor (1), poor (2), good (3), very good (4), and excellent (5). Also, any soft tissue necrosis, suppuration, and/or graft exposure were clinically evaluated at 3 and 6 months postoperatively.

#### Sensory disturbance

It was assessed by patients’ complaint (patient questionnaire) to determine any change in subjective sensation in the lower lip, chin and mental areas.

#### Radiographic evaluation

**Fig. 5:**
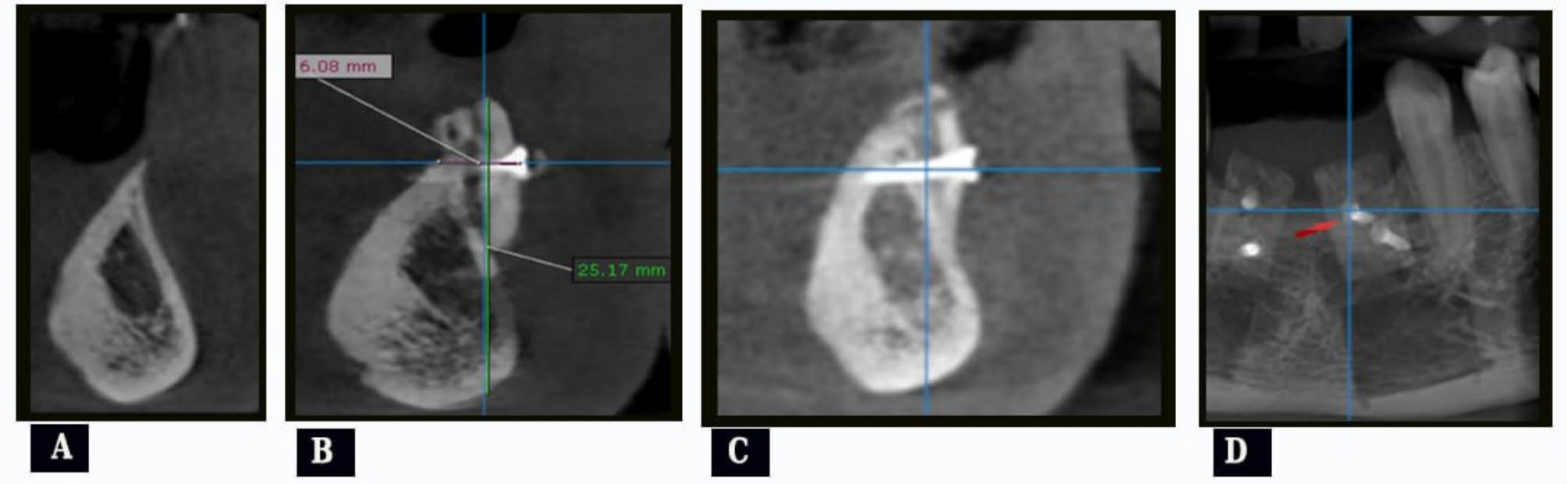
(case#3): **A**, Preoperative CBCT cross sectional view showing atrophied alveolar ridge; **B**, Immediate postoperative CBCT cross sectional view showing measurement of alveolar ridge height and width; **C**, CBCT cross sectional view showing alveolar ridge 6 months after grafting; **D**, Adaptation of the two reference lines to be crossed at the head of the coronal screw in CBCT panoramic view (arrow)

CBCT cross section view was used for evaluation of alveolar ridge width and height preoperatively (Fig. 5, A), and immediate postoperative (within one week after grafting; Fig. 5, B), and at 6months after grafting (Fig. 5, C). Horizontal and vertical bone gain was calculated by subtracting horizontal and vertical bone dimensions immediately and 6months postoperatively from preoperative ridge dimensions. Bone graft resorption at 6months was calculated by subtracting bone gain after 6months from immediate postoperative bone gain. All patient scans were taken by a Planmeca ProMax^®^ 3D unit (Planmeca OY, Helsinki, Finland) using fixed imaging parameter at every scan. All DICOM data were then analyzed using OnDemand3D software (Version 1, CyberMed, Seoul, South Korea). For standardization, the two reference lines were adapted to be crossed at the head of the coronal screw in CBCT panoramic view (arrow in Fig. 5, D) in all measurement periods.

### Statistical methods

Data was analyzed using Statistical Package for Social Science software computer program version 26 (SPSS, Inc., Chicago, IL, USA). Quantitative data was presented in mean& standard deviation. Student’s t-test(paired) was used to compare two related groups of parametric data, while repeated measures ANOVA followed by post-hoc Bonferroni test was used to compare more than two related groups. P value less than 0.05 was considered statistically significant.

### Results (Table 1)

**Table 1:** Patient gender and age; site; postoperative pain; soft tissue healing; preoperative bone width and height; horizontal and vertical bone gain and bone loss

No.	Gender/ Age (years)	Site	Pain after 3days	Soft tissue healing with Landry index^(14)^ after 14 days	Preoperative bone dimensions		Horizontal bone gain (mm)		Vertical bone gain (mm)		Bone loss (mm)	
					width	height	immediate	After 6 months	immediate	After 6 months	horizontal	Vertical
1	M/ 20	U/ Left 1	2	4	5.57	12.81	3.75	3.03	5.94	4.18	0.54 (15.12%)	1.76 (29.62%)
2	F/ 25	U/ Left 12	4	3	5.80	12.60	3.8	3.15	6.0	4.5	0.65 (17.1%)	1.5 (25%)
3	F/ 20	L/ Right 12	3	4	3.14	21.17	2.94	2.62	4	2.55	0.32 (10.88%)	1.45 (36.2%)
		L/ Left 12		5	2.62	19.72	3.46	3.30	3.46	3	0.16 (4.62%)	0.46 (13.2%)
4	F/ 20	U/ Right 2	7	4	2.80	12.80	3.7	3.3	6.4	5	0.4 (10.8%)	1.4 (21.87%)
5	F/ 19	U/ Left 1	5	5	5.10	11.20	5.10	4.4	7.60	5.8	0.7 (0.13%)	1.8 (23.68%)
6	F/ 18	U/ Right 2	4	4	3.75	13.60	3.75	3.05	5.90	4	0.70 (18.66%)	1.90 (32.20%)
		U/ Left 2		4	4	13	4	3.20	5.90	4.25	0.80 (20%)	1.65 (27.96%)
7	F/ 22	L/ Right 1	3	4	3.20	14.50	5.7	4.8	5.8	3.7	0.9 (15.78%)	2.1 (36.20%)
8	M/ 26	U/ Right 3	4	3	6.5	10.25	5.55	4.50	7.40	5.60	1.05 (18.91%)	1.8 (24.32%)
9	M/ 42	L/ Left 1	5	3	3.25	16.	3.9	2.71	9	7.2	1.19 (30%)	1.8 (20%)
10	F/ 35	U/ Right 1	3	3	4.35	11.54	3.95	2.90	7.75	6.20	1.05 (26.58%)	1.55 (20%)
		U/ Left 1		4	5	10.50	3.90	2.8	7.3	5.55	1.1 (28.20%)	1.75 (23.97%)
11	M/ 24	U/ Right 1	5	4	4.25	13.56	5.05	4.24	6.74	4.89	0.8 (15.8%)	1.85 (27.44%)
	4 males; 7 females. Age: mean ± SD = 24.63 ± 7.27	10 blocks upper anterior& 4 lower anterior	mean ± SD = 4.09 ± 1.31	Mean ± SD= 3.8 ± 0.68			mean ± SD= 4.17 ± 0.77 P1˂0.001*	mean ± SD= 3.52 ± 0.75 P2˂0.001*	mean ± SD= 6.51 ± 1.01 P3˂0.001*	mean ± SD= 4.74 ± 1.03 P4˂0.001*	mean ± SD= 0.74 ± 0.24 (17.74%)	mean ± SD= 1.62 ± 0.19 (24.88%)
											P5˂0.001*	

**Abbreviations**: M, male; F, female. U, upper anterior; L, lower anterior. Data expressed as mean ± SD; P: probability; *Significance˂0.005. P1& P3, immediate horizontal and vertical bone gain; P2& P4, horizontal and vertical bone gain after 6months; P5, vertical versus horizontal bone loss

In this study 14 L-shape bone blocks (10 and 4 bone blocks in maxilla and mandible, respectively) were grafted to 11 patients. The patients were 4males and 7females, with age ranged from 18 to 42 years (mean ± SD = 24.63 ± 7.27years).

### Regarding postoperative pain

at 3days post-surgically pain was ranged from 2 to 7 with mean ± SD = 4.09 ± 1.31.

### Regarding soft tissue healing

2weeks after surgery, soft tissue healing was uneventful for all patients with mean ± SD = 3.8 ± 0.68 on the Landry index (14). Bone block exposure about 2 mm in diameter occurred in one case at 3 months follow-up. After smoothing of the exposed bone and H2O2 mouth rinsing with local application of chlorhexidine gel for 2weeks, normal healing occurred.

### Regarding sensory disturbance

there was no sensory disturbance in the tributary of the mental nerves.

### Bone gain

#### Horizontal

Mean ± SD of horizontal bone gain was 4.17 ± 0.77 mm immediate postoperative, and was 3.52 ± 0.75 mm after 6months. There was a statistically significant increase in bone width immediate postoperative (P˂0.001) and 6months (P˂0.001) in comparison to preoperative bone width.

#### Vertical

Mean ± SD of vertical bone gain was 6.51 ± 1.01 mm immediate postoperative, and was 4.74 ± 1.03 mm after 6 months. There was a statistically significant increase in bone height immediate postoperative (P˂0.001) and 6months (P˂0.001) in comparison to preoperative bone height.

### Bone loss

Mean ± SD of horizontal and vertical bone loss were 0.74 ± 0.24 mm (17.74%) and 1.62 ± 0.19 mm (24.88%) after 6 months, respectively. At 6months postoperatively, there was a statistically significant increase in vertical bone loss compared to horizontal bone loss (P˂0.001).

## Discussion

Augmentation of a deficient alveolar ridge is essential to attain satisfactory functional and aesthetic outcomes with dental implants, particularly in the anterior maxilla and mandible. Several approaches have been proposed for 3D ridge augmentation, such as GBR, distraction osteogenesis and bone block [4, 6–8]. Nevertheless, autogenous bone grafting has been considered the gold standard and the most predictable approach for ridge reconstruction [14].

In comparison to extraoral bone grafts, intraoral grafts have faster revascularization, better graft integration, and less resorption rate [15]. Symphysis and ramus are the most common intraoral donor sites [16]. Symphyseal bone block was selected in this study due to its proximity to the recipient site, also it is corticocancellus in nature (65% cortical bone and 36% cancellous bone) enhancing a faster revascularization and healing compared with ramus bone block which is almost 100% cortical in nature [17]. In addition, the symphysis offers a greater graft thickness(5–8 mm) compared to the mandibular ramus(3–4 mm) [18], which is essential for L-shape bone block formation.

At the recipient site, a mid-crestal incision that continued in the sulcus for two teeth on either side of the defect with bilateral oblique incisions were performed in this study, rather than papilla-sparing release incisions which overlie the interface between recipient and donor bone that can result in wound dehiscence and graft exposure [18].

According to Landry et al. [19], a score of 3–5 indicates a successful soft tissue healing. In this study, soft tissue healing was uneventful for all patients with a mean score of 3.8 on Landry index [19]. There was no early tissue dehiscence or graft exposure, this may be related to adequate flap relaxation by periosteal incisions and blunt muscle dissection from the anterior maxilla and mandible having a tension free and water tight closure. In addition, artificial membranes were not used in this study, so the soft tissue flap was in contact with its natural bone surface, favoring a faster and more stable reattachment of the flap with the underlying bone block [5, 20]. However, late small (2 mm) graft exposure, after 3months, occurred in one case which may be caused by sharp edges of the graft. This graft exposure was treated by smoothing of the exposed bone and H_2_O_2_ mouth rinsing with local application of chlorhexidine gel for 2 weeks. Thoma et al. [21] observed exposure of one (8.33%) autogenous bone block 6 days post-surgery and this block failed, as antiseptic treatment was unsuccessful. Moreover, Luis et al. [22] in their systematic review reported 9.76% dehiscence rate for autogenous bone blocks whereas Felice et al. [23] documented 20% dehiscence of autogenous bone blocks. On contrary, Pistilli et al. [24] observed no dehiscence (0%) in autogenous bone blocks. Also, Cordaro et al. [3] after grafting 18 alveolar ridges with autogenous bone blocks, revealed that soft tissue healing was uneventful in all cases with no graft failure.

Khoury et al. [25] revealed exposure of inferior alveolar nerve in 4.33% of cases after harvesting bone blocks from the retromolar region leading to sensory disturbance lasting up to 6 months with minor nerve injury in 0.5% of cases represented as hypesthesia or paresthesia that persisted for 12months. Also, Silva et al. [26] in their systematic review, documented 18.57% sensory alterations of the skin and mucosa of chin after harvesting bone block from the symphysis. In contrary, in this study there was no mental nerve injury in any case, which may be related to the safety distance(5 mm) of osteotomies from the apices of the teeth and mental nerves [13], and the use of piezo-surgery in harvesting bone blocks. Due to the low frequency of the ultrasonic waves of piezoelectric device(24–29 kHz), it selectively cuts mineralized tissues without violating soft tissues as nerves, sinus membrane, and blood vessels (soft tissues can only be cut at frequencies more than 50 kHz) [27–29].

Regarding bone gain, in this study average bone gain was 4.17 mm horizontally and 6.51 mm vertically immediate postoperatively, and 3.52 mm horizontally and 4.74 mm vertically after 6months. Luis et al. [22] in their systematic review reported that horizontal bone gain was 4–5 mm, while vertical bone gain was 5.09–5.1 mm after ridge augmentation with autogenous bone blocks. Cordaro et al. [3] revealed that, after 6 months the mean lateral bone gain was 5.023 mm, while the mean vertical bone gain was 2.2 mm. Khoury et al. [2] found that, the vertical gained bone was 7.3 mm, and the horizontal bone gain was 7.7 mm in maxillary posterior ridge augmentation after 3 months using ramus bone block.

No graft failure occurred in this study, this may be attributed to the osteoconductive, osteoinductive, and osteogenic characteristics of autogenous bone graft. The osteogenic capacity of autogenous bone block enhances bone growth in between the interface of the graft and the host bone [14]. In addition, the stability of the created 3D space that results from the stiffness of the bone blocks ensures the graft healing without disruption [2]. Furthermore, perforations into underlying bone marrow accelerate revascularization and facilitate graft integration [30]. Bone marrow penetrations have several advantages as enhancing the healing process by inducing bleeding and blood clot formation, allowing progenitor cells and blood vessels to reach the bone graft site which enhances angiogenesis, and improving the physical integration of the grafted bone and the recipient site [30, 31]. In addition, using piezoelectric device in harvesting bone block may stimulate graft healing, Preti et al. [32] concluded that piezoelectric bone surgery appears to be more efficient in early bone healing compared to burs and saws, as it induces early increase in BMPs, and accelerates bone remodeling. In contrary, Luis et al. [22] in their systematic review reported a 6.1% failure rate of autogenous bone blocks, while Joshi [33] reported a 14.8% failure rate of symphyseal bone blocks.

Resorption of grafted autogenous bone is always a matter of concern. In this study horizontal bone loss was 0.74 mm (17.74%), while vertical bone loss was 1.62 mm (24.88%) after 6months. Bone resorption occurred more vertical than horizontal (P˂0.001); this can be explained by the fact that the horizontal arm of L-shape block was mainly cancellous in nature while the vertical arm was corticocancellous. In addition, the presence of fixation screws horizontally rather than vertically may decrease soft tissue pressure on the bone block labially, resulting in less horizontal bone resorption. Cordaro et al. [3] reported that mean resorption rates of symphysial bone blocks after 6months were 23.5% and 42% for horizontal and vertical grafts, respectively. Proussaefs et al. [34] reported 16.34% vertical resorption in 4–6months. They concluded that alveolar ridge vertical augmentation with autogenous bone block from the ramus is a predictable treatment option. Widmark et al. [35] documented 25% horizontal resorption after 3–5 months when the symphyseal bone block was grafted to the anterior maxilla. On the contrary, Guimaraes et al. [36] revealed a 2.6% autogenous bone block resorption rate. In addition, Thoma et al. [21] observed a relatively low resorption rate, which might be related to the coverage of the autogenous graft with DBBM particles and the relatively short evaluation period(4months). Also, Zaki et al. [37] in their systematic review and meta-analysis concluded that the use of membranes decreased the autogenous intraoral block grafts resorption.

In this study we used only high-speed surgical burs for trimming of the bone blocks. In the future studies, small bone cutting disc, suction with filter bone collector, and mini bone scraper can be used carefully to collect the bone chips that result from trimming of the bone blocks which can be used to fill the gap between the graft and underlying alveolar bone.

### Limitations of the study

Small sample size, short period of follow-up, lack of control group, invasive method of preparing the graft, and second surgical site were limitations of this study.

## Conclusion

Within the limitation of this study, it was concluded that using L- shape autogenous bone block harvested from the symphysis for alveolar ridge augmentation is a safe, predictable and effective method for 3D ridge augmentation.

## Data Availability

The author confirms that all data generated or analysed during this study are included in this published article. Any further data are available from the authors upon reasonable request.
